# Structure of a Vaccine-Induced, Germline-Encoded Human Antibody Defines a Neutralizing Epitope on the SARS-CoV-2 Spike N-Terminal Domain

**DOI:** 10.1128/mbio.03580-21

**Published:** 2022-04-25

**Authors:** Clara G. Altomare, Daniel C. Adelsberg, Juan Manuel Carreno, Iden A. Sapse, Fatima Amanat, Ali H. Ellebedy, Viviana Simon, Florian Krammer, Goran Bajic

**Affiliations:** a Department of Microbiology, Icahn School of Medicine at Mount Sinai, New York, New York, USA; b Graduate School of Biomedical Sciences, Icahn School of Medicine at Mount Sinai, New York, New York, USA; c Department of Pathology and Immunology, Washington University School of Medicine, St. Louis, Missouri, USA; d Andrew M. and Jane M. Bursky Center for Human Immunology and Immunotherapy Programs, Washington University School of Medicine, St. Louis, Missouri, USA; e Center for Vaccines and Immunity to Microbial Pathogens, Washington University School of Medicine, St. Louis, Missouri, USA; f Division of Infectious Diseases, Department of Medicine, Icahn School of Medicine at Mount Sinai, New York, New York, USA; g The Global Health and Emerging Pathogens Institute, Icahn School of Medicine at Mount Sinai, New York, New York, USA; h Department of Pathology, Molecular, and Cell-Based Medicine, Icahn School of Medicine at Mount Sinai, New York, New York, USA; Duke University School of Medicine

**Keywords:** adaptive immunity, coronavirus, electron microscopy, monoclonal antibodies, vaccine

## Abstract

Structural characterization of infection- and vaccination-elicited antibodies in complex with antigen provides insight into the evolutionary arms race between the host and the pathogen and informs rational vaccine immunogen design. We isolated a germ line-encoded monoclonal antibody (mAb) from plasmablasts activated upon mRNA vaccination against severe acute respiratory syndrome coronavirus 2 (SARS-CoV-2) and determined its structure in complex with the spike glycoprotein by electron cryomicroscopy (cryo-EM). We show that the mAb engages a previously uncharacterized neutralizing epitope on the spike N-terminal domain (NTD). The high-resolution structure reveals details of the intermolecular interactions and shows that the mAb inserts its heavy complementarity-determining region 3 (HCDR3) loop into a hydrophobic NTD cavity previously shown to bind a heme metabolite, biliverdin. We demonstrate direct competition with biliverdin and that, because of the conserved nature of the epitope, the mAb maintains binding to viral variants B.1.1.7 (alpha), B.1.351 (beta), B.1.617.2 (delta), and B.1.1.529 (omicron). Our study describes a novel conserved epitope on the NTD that is readily targeted by vaccine-induced antibody responses.

## INTRODUCTION

Severe acute respiratory syndrome coronavirus 2 (SARS-CoV-2) has officially caused more than 185 million infections and more than 4 million official deaths worldwide (World Health Organization). Immune responses mounted upon COVID-19 (coronavirus disease 2019) have been a subject of active investigations by many groups. As safe and effective vaccines are developed and administered in record time (1, 2), there is an urgent need to better understand the quality of the vaccine-induced immune responses, the broadly neutralizing epitopes targeted, and their effectiveness against newly emerging, potentially more transmissible viral variants. Understanding the immunodominance landscape of the major antibody target, the spike glycoprotein, at a structural level will identify the requirements for broader SARS-CoV-2 antibody responses and provide the foundation for developing the next generation of vaccines.

The viral spike glycoprotein is both the attachment factor that binds angiotensin-converting enzyme 2 (ACE2) on host cells and the viral fusogen that mediates the fusion of the viral membrane with that of the host cell (3). The fusion step depends on furin-mediated cleavage, resulting in the generation of N-terminal S1 and C-terminal S2 domains (4). The second, subsequent cleavage of S2 is mediated by a serine protease, TMPRSS2, or by cathepsins (5). The spike glycoprotein is the main target of neutralizing antibody responses and, hence, the focus of most vaccines. Antibody responses to natural infection in the serum, in the memory B cell compartment, and, to a lesser degree, at mucosal surfaces against spike have been well characterized in terms of kinetics, binding specificity, and neutralization potency (6–18). Anti-SARS-CoV-2 spike serum antibody titers after natural infection are variable, may decline to some degree over time (17, 19), and have suboptimal neutralization activity against more recent viral variants despite being protective (20, 21). Antibodies derived from memory B cells target both unique and, to a certain extent, overlapping epitopes that contribute to polyclonal epitopic coverage of spike and ensure preserved binding to viral variants of concern (VOCs) (22–29). We have recently shown that immunization with mRNA vaccines results in antibodies targeting not only the receptor binding domain (RBD) but also the N-terminal domain (NTD) (30).

We previously identified a neutralizing monoclonal antibody (mAb), PVI.V6-14, derived from the plasmablast response mounted by a naive study participant after two doses of an mRNA vaccine whose heavy and light chains both contained no somatic hypermutation (30). In the present study, we focus on the early events of B cell activation after SARS-CoV-2 vaccination to structurally profile novel antibody epitopes. We determined the structure of PVI.V6-14 Fab in complex with the SARS-CoV-2 spike at a 3.6-Å resolution by single-particle electron cryomicroscopy (cryo-EM) and showed that it bound a lateral side of the NTD. The interaction was mediated mainly by the heavy complementarity-determining region 3 (HCDR3) loop, with minimal contacts from the light chain. We found that mAb PVI.V6-14 belongs to an as-yet-undescribed class of antibodies that bind within a hydrophobic cavity, previously identified to bind a heme metabolite, biliverdin (31). Our functional binding and neutralization data confirm that the antibody competes with biliverdin and also underscore the antibody’s capacity to recognize emerging viral variants of concern. Our study puts forward a concept for a therapeutic combination antibody cocktail that comprises both RBD- as well as NTD-neutralizing antibodies. Collectively, our results inform on the rational design of a novel class of immunogens for next-generation vaccines that provide broad protection against currently circulating as well as future, newly emerging SARS-CoV-2 variants of concern.

## RESULTS

### Neutralizing antibody PVI.V6-14 binds a lateral cavity in the NTD.

**FIG 1:**
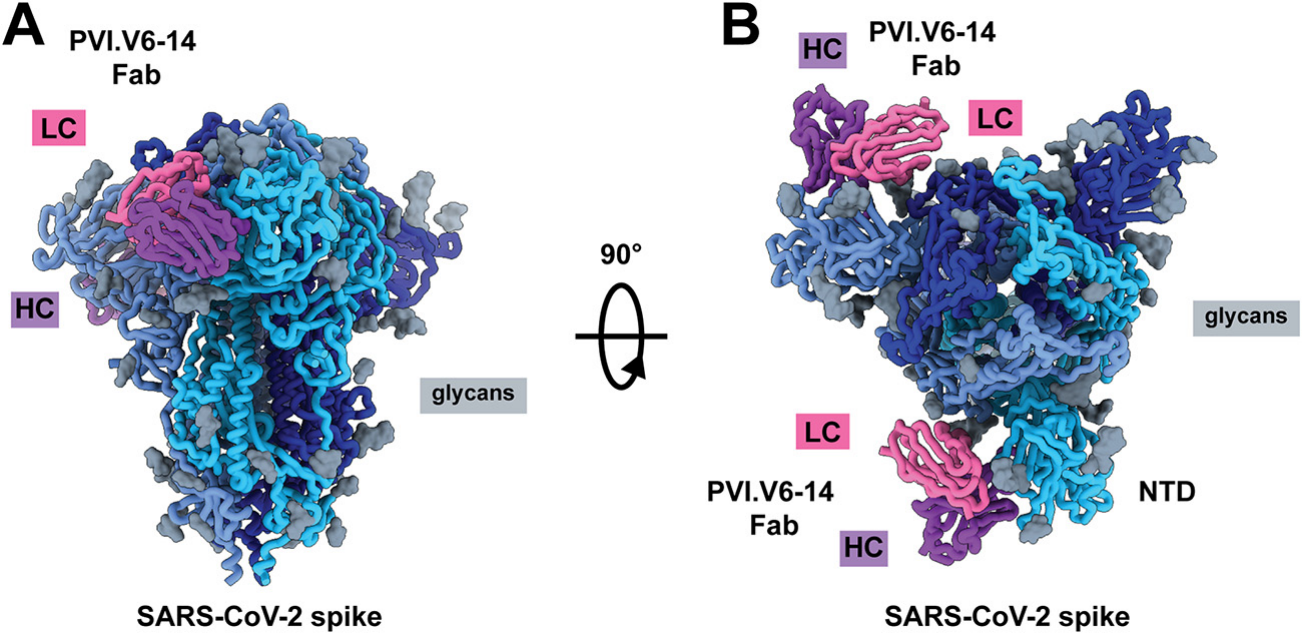
PVI.V6-14 mAb recognizes a novel epitope on the SARS-CoV-2 spike NTD. Shown is a cryo-EM structure model of the SARS-CoV-2 spike trimer (the three protomers are in hues of blue, and glycans are in gray) with PVI.V6-14 Fab (the heavy chain [HC] is in purple, and the light chain [LC] is in pink) bound to the NTD (A), with a 90°-rotated view (B). Two Fabs are bound per trimer in the final reconstruction.

We previously reported the isolation and characterization of plasmablast-derived antibodies from individuals who received the Pfizer/BioNTech mRNA SARS-CoV-2 vaccine BNT162b2 (30). We noted that the overall neutralizing antibody responses were directed toward both the RBD and NTD, indicating the codominance of these two spike domains. Since the NTD emerges to be an important component of the vaccine-induced responses, we wanted to expand on our understanding of neutralizing epitopes in this region of the SARS-CoV-2 spike for which only limited structural information is currently available. We therefore focused our attention on participant V6, who mounted a strong neutralizing antibody response to the NTD region of spike upon vaccination. We selected, for further analysis, a neutralizing antibody, PVI.V6-14, that bound to the NTD and whose V(D)J sequence was identical to that of the germ line. Indeed, PVI.V6-14’s heavy chain variable (V_H_) sequence is identical to the human IGHV4-39*01 germ line sequence (see Fig. S2A in the supplemental material). The rearranged V(D)J complementarity-determining region 3 (CDR3), which encodes 21 amino acid residues, is composed of IGHD3-10*01 and IGHJ4*02 (Fig. S2B). The kappa light chain was also unmutated and encoded by IGHKV1-12*01 (Fig. S2C and D). We determined its structure in complex with the spike glycoprotein at a 3.6-Å nominal resolution using single-particle cryo-EM (Fig. 1, Fig. S1 and S3, and Table S1). PVI.V6-14 binds the NTD on the side that is anticlockwise looking down on RBDs and perpendicular to the 3-fold axis of the spike (Fig. 1A and B and Fig. S1A and B). There are two Fabs bound per spike trimer in the final cryo-EM reconstruction. Incidentally, RBDs are in the “down” configuration on the two protomers whose NTDs are in complex with Fab; the unbound protomer has its RBD partially “up.” Our NTD-Fab-focused, locally refined map shows that the heavy chain CDR3 loop protrudes deep into the NTD cavity (Fig. S1C and D). PVI.V6-14 appears to stabilize the NTD as the cryo-EM map shows a clear, undisrupted volume for the entire domain, allowing us to trace the complete polypeptide chain with high confidence (Fig. S1C).

10.1128/mbio.03580-21.1 FIG S1 PVI.V6-14 mAb recognizes a novel epitope on the SARS-CoV-2 spike NTD. (A and B) Cryo-EM reconstruction at 3.6 Å of the SARS-CoV-2 spike trimer (shades of blue) fit into the cryo-EM volume (gray) with PVI.V6-14 Fab (the heavy chain is in purple, and the light chain is in pink) bound to the NTD, with a 90°-rotated view along the 3-fold axis. Two Fabs are bound, and two RBDs are in the “down” conformation. (C and D) Atomic model of the NTD–PVI.V6-14 V_H_V_L_ fit into the focus-refined map at a 3.7-Å nominal resolution, with HCDR3 inserted into the hydrophobic NTD pocket. Download FIG S1, TIF file, 1.5 MB. Copyright © 2022 Altomare et al. 2022 Altomare et al. https://creativecommons.org/licenses/by/4.0/ This content is distributed under the terms of the Creative Commons Attribution 4.0 International license.

10.1128/mbio.03580-21.2 FIG S2 PVI.V6-14 is an unmutated rearranged V(D)J sequence. (A) Nucleotide sequence alignment of the PVI.V6-14 variable heavy gene (IGHV) with its germ line IGHV4-39*01. (B) V, D, and J gene fragments mapped onto the nucleotide sequence of the PVI.V6-14 HCDR3 loop. The translated amino acid sequence is shown below. (C) Nucleotide sequence alignment of the PVI.V6-14 variable kappa light gene (IGKV) with its germ line IGKV1-12*01. (D) V and J gene fragments mapped onto the nucleotide sequence of the PVI.V6-14 LCDR3 loop. The corresponding amino acid sequence is shown below. Download FIG S2, TIF file, 0.9 MB. Copyright © 2022 Altomare et al. 2022 Altomare et al. https://creativecommons.org/licenses/by/4.0/ This content is distributed under the terms of the Creative Commons Attribution 4.0 International license.

10.1128/mbio.03580-21.3 FIG S3 Cryo-EM data processing scheme and local resolution estimation of the final map. (A and B) Representative electron micrograph (A) and 2D class averages (B) obtained for the SARS-CoV-2 spike ectodomain in complex with PVI.V6-14 Fab. (C and D) Local resolution and gold-standard Fourier shell correlation curves calculated with cryoSPARC v3.3.1. for the overall (C) and locally refined (D) NTD-Fab complex. Both maps are sharpened. Shown are details of HCRD3 binding to the NTD with the cryo-EM volume (contour level of 0.1). (E) Detailed cryo-EM data processing workflow. See Materials and Methods and Table S1 in the supplemental material for more details. Download FIG S3, TIF file, 1.3 MB. Copyright © 2022 Altomare et al. 2022 Altomare et al. https://creativecommons.org/licenses/by/4.0/ This content is distributed under the terms of the Creative Commons Attribution 4.0 International license.

10.1128/mbio.03580-21.5 TABLE S1 Cryo-EM data collection and model validation statistics. Download Table S1, DOCX file, 0.02 MB. Copyright © 2022 Altomare et al. 2022 Altomare et al. https://creativecommons.org/licenses/by/4.0/ This content is distributed under the terms of the Creative Commons Attribution 4.0 International license.

**FIG 2:**
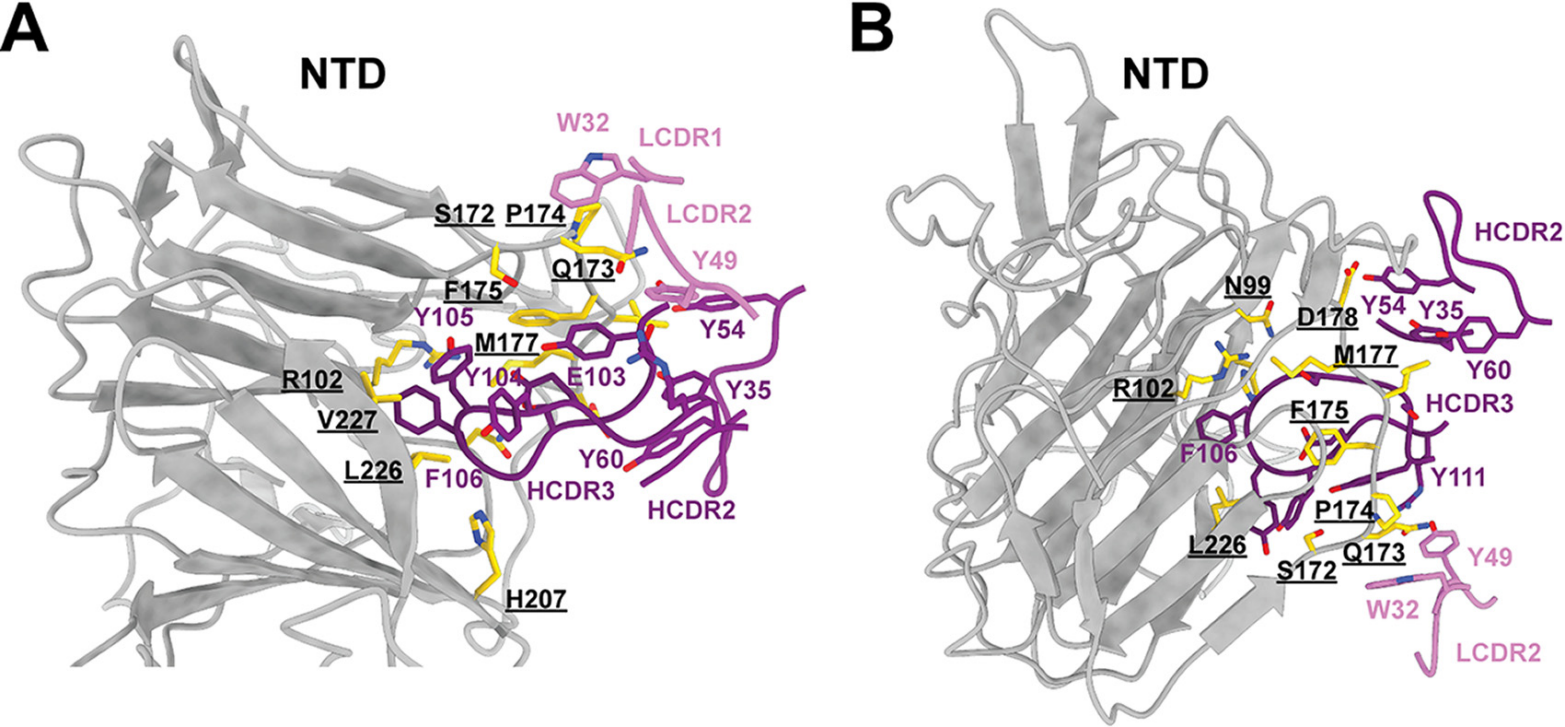
PVI.V6-14 mAb binds the spike NTD primarily through its HCDR3 loop. Shown are two different views of the details of the intermolecular interactions between PVI.V6-14 and the NTD that are dominated by the HCDR3 loop. NTD-interacting amino acid residues are shown in gold, with residue number labels underscored.

PVI.V6-14 binds on the NTD between two glycans, N122 and N282, and contacts the hydrophobic cavity primarily through its HCDR3 loop (Fig. S1C and D) composed of a string of aromatic amino acid residues, Tyr104, Tyr105, and Phe106 (Fig. 2A and B). In particular, Tyr105 interacts with Arg190, Phe192, and His207 in the NTD. Additional HCDR3 contacts are provided by Glu103 and Ser108 that form a hydrogen bond donor and acceptor, respectively, and interact with Gln173 and Pro174 (Fig. 2A and B). V_H_ makes additional contacts with the NTD through HCDR1 Tyr35 and HCDR2 Tyr54 and Tyr60. Minor light chain contacts are supplied by light complementarity-determining region 1 (LCDR1) Trp32, which pi-pi stacks against Pro174 on the NTD, and LCDR2 Tyr49, which hydrogen bonds with Gln173 (Fig. 2A and B).

### PVI.V6-14 binds viral variants of concern.

**FIG 3:**
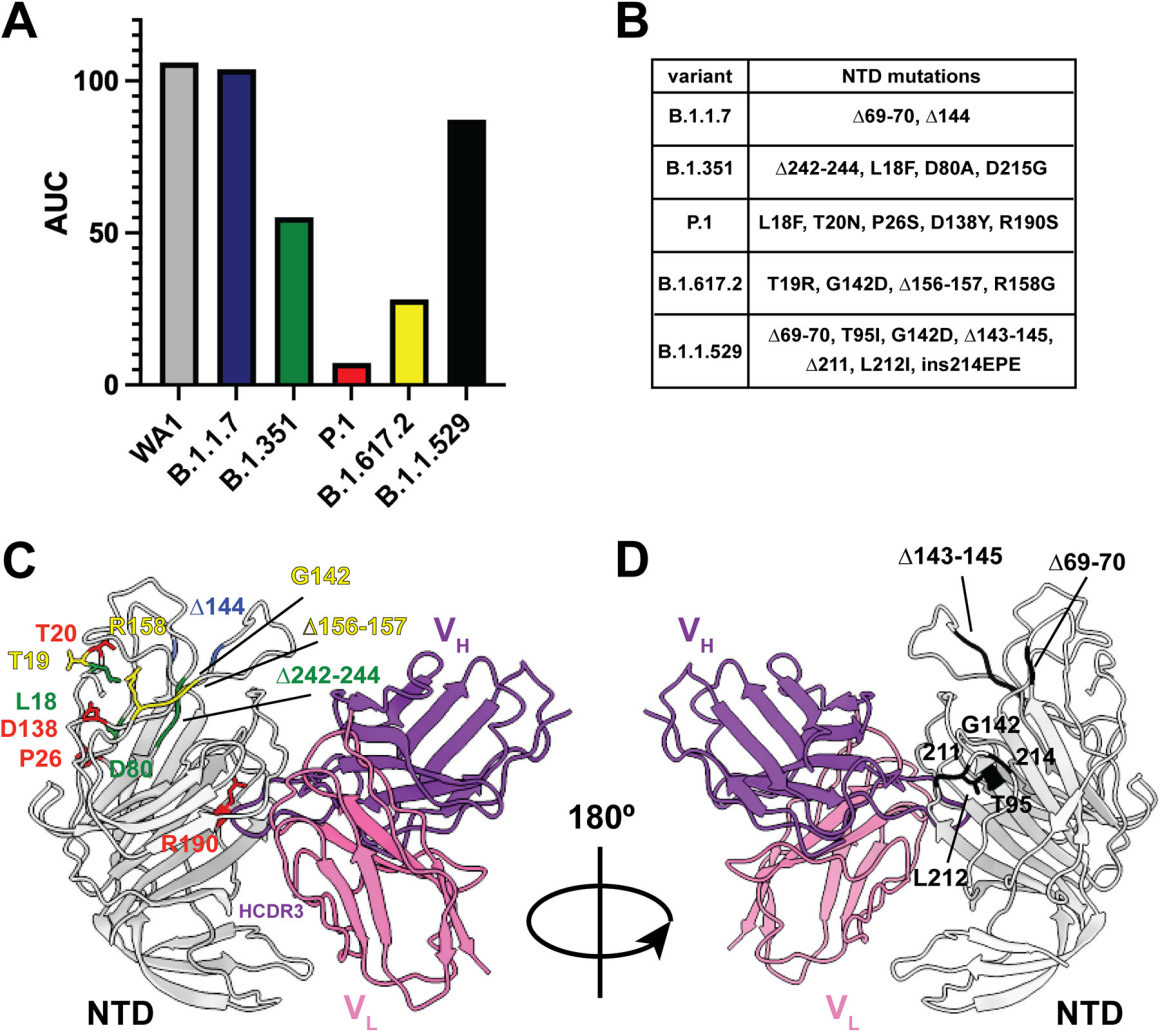
PVI.V6-14 binds viral variants of concern B.1.1.7 (alpha), B.1.351 (beta), B.1.617.2 (delta), and B.1.1.529 (omicron) but not P.1 (gamma). (A) Binding of PVI.V6-14 IgG to WA1/2020, (gray), B.1.1.7 (alpha) (blue), B.1.351 (beta) (green), P.1 (gamma) (red), B.1.617.2 (delta) (yellow), and B.1.1.529 (omicron) (black) NTDs of SARS-CoV-2 spike. The area under the curve (AUC) was calculated by subtracting the average of blank values plus 3 times the standard deviation of the blank values. Shown are means from a representative experiment performed in triplicates. Anti-polyhistidine IgG was used as positive control across the ELISA plates. (B) Table recapitulating the VOC amino acid mutations. (C and D) Structural mapping of the VOC mutations onto the NTD in complex with PVI.V6-14 Fab. The structure explains the mAb dependence on the R190 residue in the NTD and the diminished binding to the P.1 VOC. The color scheme is the same as the one described above for panel A.

We next performed a sequence alignment of the spike proteins of 35 different sarbecoviruses and noticed that the NTD cavity was comprised of amino acid residues that were conserved across SARS-CoV-2 isolates and bat coronaviruses with pandemic potential (Fig. S4). We hypothesized that PVI.V6-14 would also bind to the emerging variants of concern (VOCs). Indeed, this antibody bound to the spike NTD of B.1.1.7 with a capacity comparable to that of the parental WA1 isolate, but it bound B.1.351, B.1.617.2, and B.1.1.529 NTDs at reduced capacities of 50%, 25%, and 90% of the initial WA1 binding, respectively (Fig. 3A and B). We note that PVI.V6-14 almost completely lost the binding to the P.1 variant (Fig. 3A and B). We next mapped the mutations of the viral variants of concern onto the NTD region within the structure of our complex (Fig. 3C and D). The structure supports the lack of an impact of the substitutions and deletions specific for the B.1.1.7 (alpha), B.1.351 (beta), B.1.617.2 (delta), and B.1.1.529 (omicron) lineages. The structure shows that the only amino acid residue substitution in P.1 close to the antibody epitopes is the arginine at position 190. This arginine residue is not directly contacted by the antibody HCDR3 loop, which suggests that, perhaps, indirect effects on the conformations of the NTD loops might be caused by the R190S substitution. We performed a sequence alignment of a select but diverse set of spike sequences across the sarbecovirus subgenus and mapped their amino acid conservation onto the structure of the NTD bound with PVI.V6-14 (Fig. S4). We note that the NTD pocket engaged by PVI.V6-14 is conserved within the sarbecovirus subgenus, which helps explain the antibody’s binding breadth. In our previous study, we noticed that PVI.V6-14 did not neutralize B.1.1.7 and B.1.351 viruses (30). Our NTD structure, therefore, offers a molecular rationale for the antibody’s breadth.

10.1128/mbio.03580-21.4 FIG S4 NTD conservation map. Shown is amino acid sequence conservation of a representative set of spike sequences from the sarbecovirus subgenus aligned and mapped onto the structure of the NTD–PVI.V6-14 complex in a top view (A) and a bottom view (B). The hydrophobic cavity contains conserved residues. Accession numbers used are as follows: NCBI accession numbers KY417152, KY417151, MK211376, KC881006, KF367457, KT444582, KY417150, NC_004718, AY304486, AY304488, and AY572034 and GISAID Epi-Cov accession numbers EPI_ISL_412860, EPI_ISL_1699444, EPI_ISL_1699443, EPI_ISL_1699446, EPI_ISL_1699445, EPI_ISL_804222, EPI_ISL_410540, EPI_ISL_412977, EPI_ISL_402125, EPI_ISL_402131, EPI_ISL_1699447, EPI_ISL_410542, EPI_ISL_410541, EPI_ISL_410544, EPI_ISL_410538, EPI_ISL_1699448, EPI_ISL_410543, EPI_ISL_1699449, EPI_ISL_410539, and EPI_ISL_410721. Download FIG S4, TIF file, 1.0 MB. Copyright © 2022 Altomare et al. 2022 Altomare et al. https://creativecommons.org/licenses/by/4.0/ This content is distributed under the terms of the Creative Commons Attribution 4.0 International license.

### Antibody PVI.V6-14 directly competes with and is inhibited by biliverdin.

**FIG 4:**
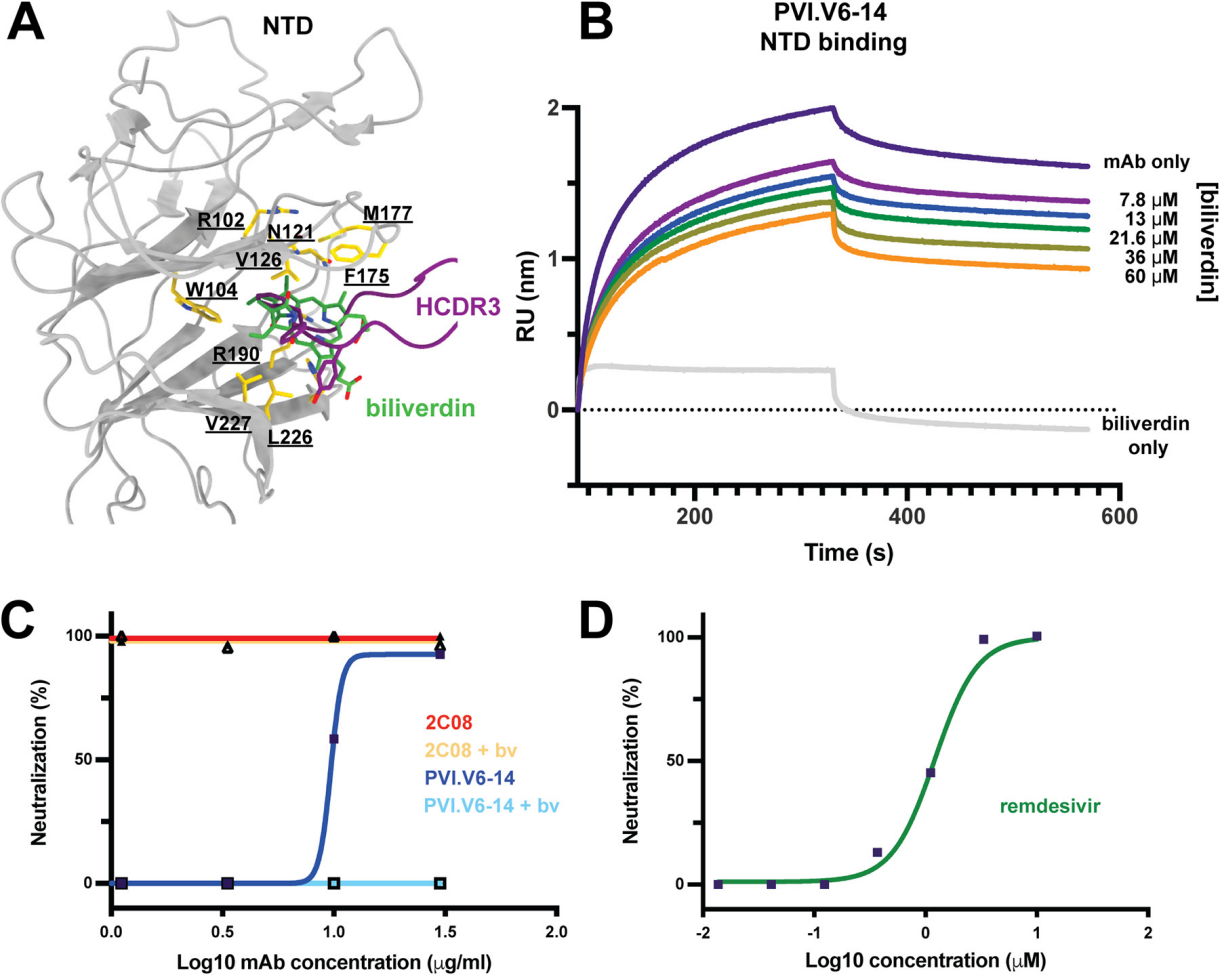
Biliverdin directly competes with PVI.V6-14. (A) Structural superposition of the biliverdin-bound NTD (PDB accession number 7B62) with the PVI.V6-14-bound NTD (this study) (PDB accession number 7RBU). Biliverdin is shown in green, and the PVI.V6-14 HCDR3 loop is shown in purple sticks. NTD-interacting amino acid residues are shown in gold, with residue number labels underscored. (B) Biolayer interferometry (BLI)-based competition assay of biliverdin with PVI.V6-14 on the recombinant NTD. RU, response units. (C) Neutralization assay with an authentic SARS-CoV-2 isolate of NTD binding PVI.V6-14 and RBD binding 2C08 mAb with and without biliverdin (bv). PVI.V6-14 directly competes with biliverdin, while 2C08 neutralization activity is unaffected by biliverdin. (D) Remdesivir neutralization control.

A previous study reported the structure of a heme metabolite, biliverdin, bound within the hydrophobic NTD pocket (31). We superposed the structure of our Fab-bound NTD with that of the biliverdin-bound one and found that our antibody, through its HCDR3 aromatic amino acid residues, was a molecular mimic of the tetrapyrrole molecule (Fig. 4A). Indeed, the two complexes share most of the NTD contact residues. To corroborate this observation, we performed a biolayer interferometry (BLI)-based competition assay with PIV.V6-14 and biliverdin (Fig. 4B). We observed concentration-dependent inhibition of PVI.V6-14 binding to the recombinant NTD by biliverdin. We next asked if biliverdin could interfere with the binding of this antibody class to a virus and performed neutralization assays on an authentic SARS-CoV-2 isolate (Fig. 4C and D). We found that biliverdin abrogated PVI.V6-14 neutralization at high concentrations (Fig. 4C). The binding of an RBD antibody, 2C08 (32), was unaffected (Fig. 4C). A remdesivir control is shown in Fig. 4D.

### PVI.V6-14 engages a distinct epitope.

**FIG 5:**
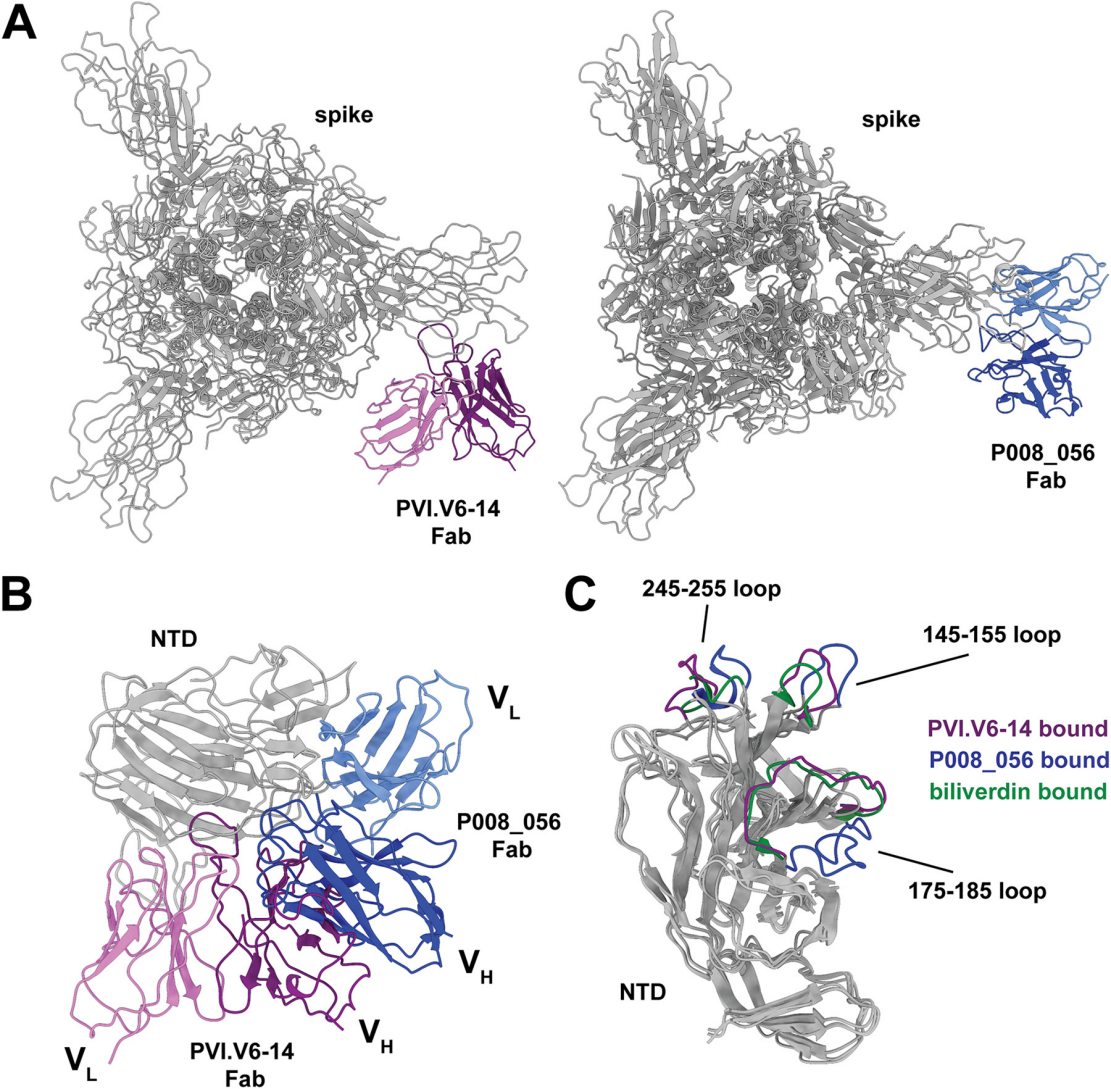
Structural comparison of PVI.V6-14 and P008_056. (A) Atomic models of the PVI.V6-14- and P008_056-bound SARS-CoV-2 spike. While both Fabs compete with biliverdin, their epitopes and angles of approach are different. (B) Structural superposition of PVI.V6-14 and P008_056 Fab-NTD complexes showing that the binding of the two classes of antibodies is mutually exclusive. (C) Structural superposition of the PVI.V6-14 (purple)-, P008_056 (blue)-, and biliverdin (green)-bound NTD. Major structural rearrangements are localized to the 245–255, 145–155, and 175–185 loops. The PVI.V6-14 NTD conformation is similar to the biliverdin-bound one.

A recent study reported the structure of an NTD-targeting antibody, P008_056, that also competes with biliverdin for binding to the NTD (31). This antibody, however, does so allosterically, while PVI.V6-14 is a direct competitor. Indeed, the two antibodies approach the spike NTD from different angles (Fig. 5A). The binding of the two antibodies to the NTD is, however, mutually exclusive for two reasons: (i) there would be a severe steric clash of the two V_H_ domains (Fig. 5B), and (ii) the NTD configuration must be “open” for P008_056 to bind and “closed” for PVI.V6-14 (Fig. 5C). Indeed, the PVI.V6-14-bound NTD structure resembles more the biliverdin-bound one, whereas the P008_056 complex induces a conformational change that reconfigures the loop from positions 175 to 185 (175–185 loop) in a downward and outward direction (Fig. 5C).

## DISCUSSION

The establishment of specific and durable humoral immunity relies on the activation of specific B cell populations that respond to infection or vaccination. Plasmablasts are a part of early responses, and their immunoglobulin repertoire most likely reflects the composition of the early antigen-specific polyclonal serum. Understanding the specificities and the neutralization potential of these responses is therefore of paramount interest for infectious disease research and vaccinology. Additionally, the quality and the distribution of epitopes targeted may differ between natural infection and vaccination. We previously reported that infection with SARS-CoV-2 results in varying levels of antibody titers against the spike glycoprotein but that mRNA vaccination, in contrast, induces consistently high titers (1, 2, 30). We also found that vaccine-derived antibody responses target more nonneutralizing epitopes than the responses from COVID-19 survivors (30). We therefore wanted to define the epitopes targeted by vaccine-induced early plasmablast responses. We specifically focused on non-RBD-directed neutralizing antibodies and selected for structural characterization PVI.V6-14, an antibody that neutralized the authentic SARS-CoV-2 isolate, albeit not very potently, whose amino acid sequence indicated that it was derived from an unmutated plasmablast.

We determined the structure of PVI.V6-14 in complex with the spike glycoprotein and showed that the antibody targets a hitherto-uncharacterized epitope. The antibody bound on the side of the NTD, away from the RBD, suggesting that the mechanism by which it neutralizes the virus is more complex than simple steric hindrance of ACE2 receptor binding. Our structure showed that PVI.V6-14 stabilized the NTD by inserting its HCDR3 loop into a hydrophobic pocket between two beta sheets. Indeed, this stabilization of the NTD allowed us to obtain a high-resolution cryo-EM reconstruction and trace the entire N-terminal domain polypeptide chain.

Our data show that PVI.V6-14 bound the NTD in a configuration that is inconsistent with the binding of another NTD antibody class (31) that allosterically competes with a heme metabolite, biliverdin, for NTD binding. PVI.V6-14, however, also competes with biliverdin, and its neutralization potency is reduced in the presence of biliverdin. Whether biliverdin or its metabolites have any biological relevance in the infectious cycle of the virus remains to be explored. A previous study suggested that SARS-CoV-2 coopted biliverdin to evade antibody responses directed to this hydrophobic cavity (31). Our data, however, show that mRNA SARS-CoV-2 vaccination induced antibody responses to this particular epitope, indicating that under physiological processes of antigen presentation to B cells, this epitope was, at least partially, unoccupied.

The emergence of viral variants of concern that contain multiple substitutions and deletions in the NTD indicates that the NTD, like the RBD, is under immune pressure. PVI.V6-14 binds a relatively conserved epitope since the antibody maintained binding to the B.1.1.7 (alpha), B.1.351 (beta), B.1.617.2 (delta), and B.1.1.529 (omicron) lineages. Binding to P.1 (gamma), which carries an R190S substitution in this pocket, was, in contrast, almost completely lost (30). However, we have shown previously that mAb PVI.V6-14 also loses neutralizing activity against B.1.1.7 and B.1.351. Should antibodies of this class also be present in the memory B cell compartment, they could potentially return to germinal centers for somatic hypermutation and affinity maturation against P.1 or related viruses with mutations within the hydrophobic pocket to gain breadth and neutralization potency. This is particularly plausible in light of the germ line nature of PVI.V6-14. Thus, targeting the NTD offers an alternative to RBD-centric vaccine immunogen design (33) and paves the way to next-generation vaccines that target the NTD in addition to the RBD and potentially to antibody therapeutics that combine both RBD- and NTD-targeting neutralizing mAbs.

## MATERIALS AND METHODS

### Protein expression and purification.

All recombinant proteins were produced using Expi293F cells (Life Technologies). Spike proteins for enzyme-linked immunosorbent assays (ELISAs) were cloned into a mammalian expression vector, pCAGGS, as described previously (34, 35) and purified after transient transfections with each plasmid. Six hundred million Expi293F cells were transfected using the ExpiFectamine 293 transfection kit and purified DNA. Supernatants were collected on day 4 posttransfection and centrifuged at 4,000 × *g* for 20 min, and finally, the supernatant was filtered using a 0.22-μm filter. Ni-nitrilotriacetic acid (NTA) agarose (Qiagen) was used to purify the protein via gravity flow, and proteins were eluted as previously described (34, 35). The buffer was exchanged using Amicon centrifugal units (EMD Millipore), and all recombinant proteins were finally resuspended in phosphate-buffered saline (PBS). Proteins were also run on sodium dodecyl sulfate (SDS)-polyacrylamide gels (5 to 20% gradient; Bio-Rad) to check for purity (36, 37). For cryo-EM, SARS-CoV-2 HexaPro spike was used (38). The protein was transiently expressed in Expi293F cells (Thermo Fisher). At 5 to 7 days posttransfection, the supernatants were harvested by centrifugation and further purified using immobilized-metal affinity chromatography (IMAC) with cobalt-Talon resin (TaKaRa), followed by a Superdex 200 Increase 10/300 GL size exclusion column (GE Healthcare).

### Biolayer interferometry.

Biolayer interferometry (BLI) experiments were performed using the BLItz system (fortéBIO, Pall Corporation). The recombinant SARS-CoV-2 NTD was immobilized on a Ni-NTA biosensor, mAb PVI.V6-14 was then applied at 2.9 μM to obtain binding affinities, and biliverdin was titrated from 60 to 7.7 μM. All measurements were repeated in subsequent independent experiments. Equilibrium dissociation constant (*K_D_*) values were obtained through the local fit of the curves by applying a 1:1 binding isotherm model using vendor-supplied software. All experiments were performed in Tris-HCl (pH 7.5) at room temperature (RT).

### ELISA.

Twenty-five nanograms of the SARS-CoV-2 spike proteins was adhered to high-capacity-binding, 96-well plates (Corning) overnight in PBS. Plates were blocked with 5% bovine serum albumin (BSA) in PBS containing Tween 20 (PBS-T) for 1 h at RT. The blocking solution was discarded, 3-fold dilutions of mAb PVI.V6-14 in PBS were added to wells, and the plates were incubated for 1 h at RT. The plates were then washed three times with PBS-T. Anti-human IgG-biotin (Abcam) in PBS-T was added to each well, and the plates were incubated for 1 h at RT. The plates were then washed three times with PBS-T. Streptavidin-horseradish peroxidase (HRP) (Abcam) in PBS-T was added to each well, and the plates were incubated for 1 h at RT. The plates were then washed three times with PBS-T. Plates were developed using the 1-step Ultra 3,3′,5,5′-tetramethylbenzidine (TMB) substrate (Thermo Fisher), stopped with sulfuric acid, and immediately read using a plate reader at 450 nm. Data were plotted in Prism 9 (GraphPad Software), and the area under the curve (AUC) was calculated.

### Neutralization assays.

Twenty thousand cells in 100 μL per well were seeded onto sterile 96-well cell culture plates 1 day prior to the neutralization assay. In general, cells were used at 90% confluence to perform the assay. Serial dilutions of the mAb samples were made in 1× minimal essential medium (MEM; Life Technologies) starting at 30 μg/mL. All work with authentic SARS-CoV-2 (isolate USA‐WA1/2020) was done in a biosafety level 3 (BSL3) laboratory according to institutional biosafety guidelines and was described in much greater detail previously (34, 39). An authentic SARS-CoV-2 isolate (USA‐WA1/2020, catalog number NR-52281; BEI Resources) was preincubated with 25 μM biliverdin for 20 min in 1× MEM. One thousand median tissue culture infectious doses (TCID_50_s) of authentic virus were added to each mAb sample (with or without biliverdin), and the mixture was incubated for 1 h inside the biosafety cabinet. Medium from the cells was removed, and 120 μL of the virus-mAb (with or without biliverdin) mixture was added to the cells for 1 h at 37°C. After 1 h, the mixture was removed, and 100 μL of each corresponding dilution was added to every well. In addition, 100 μL of 1× MEM was also added to every well. Cells were incubated for 48 h at 37°C, after which the medium was removed and 150 μL of 10% formaldehyde (Polysciences) was added to inactivate the virus. For the assay control, remdesivir was used at 10 μM. After 24 h, cells were permeabilized and stained using an antinucleoprotein antibody, 1C7, as discussed in detail previously (34, 40).

### Cryo-EM sample preparation and data collection.

SARS-CoV-2 spike HexaPro was incubated with PVI.V6-14 Fab at 1 mg/mL at a molar ratio of Fab to spike of 1.5:1 for 20 min at 4°C. Three-microliter aliquots of the spike-Fab complex were applied to UltrAuFoil gold R0.6/1 grids, subsequently blotted for 3 s at blot force 3 at 20°C with 100% humidity, and then plunge‐frozen in 100% liquid ethane using an FEI Vitrobot Mark IV system. Grids were imaged on a Titan Krios microscope operated at 300 kV and equipped with a 15-eV energy filter and a Gatan K3 Summit direct detector. A total of 10,690 movies were collected in superresolution counting mode at 15 e^−^/pixel/s for 4.03 s for a total dose of 50 e^−^/Å^2^/s. Images were collected at a magnification of ×81,000, corresponding to a calibrated pixel size of 1.12 Å/pixel, with a 0.56-Å/pixel superresolution with a defocus range of −2.5 to −0.8 μm.

### Cryo-EM data processing.

Data processing was done using Relion (41). A total of 9,367 micrographs were aligned and dose weighted using Relion’s implementation of MotionCorr2 (42). The contrast transfer function (CTF) estimation was calculated using GCTF (43). Particles were picked with Topaz (44) with a model trained with a subset of refined, classified particles picked using crYOLO (45) with a particle diameter value of 330 Å. A total of 759,324 picked particles were binned to ∼12 Å/pixel and subjected to reference-free two-dimensional (2D) classification. A total of 179,294 selected particles were then extracted to ∼6 Å/pixel and subjected to a second round of 2D classification. A total of 88,192 selected particles were then subjected to one round of three-dimensional (3D) classification with 50 iterations (angular sampling at 7.5° for 50 iterations followed by 0.9° with a local search for 25 iterations) using Relion and a previously processed spike-Fab complex as a reference, yielding 60,639 particles in the final subset. Particles were then extracted to 1.12 Å/pixel, aligned using 3D auto-refine, and then unbinned to 0.56 Å/pixel for another round of 3D auto-refine in Relion. The unbinned particles were imported into cryoSPARC (46) for one round of nonuniform refinement with per-particle defocus refinement without imposed symmetry, yielding the final global map at a nominal resolution of 3.6 Å. Map sharpening of the global reconstruction was performed in cryoSPARC. The protomer with the best Fab density was subjected to local refinement using cryoSPARC with a soft mask extended by 12 pixels and padded by 30 pixels encompassing the NTD and Fab. This yielded the final local map at a 3.7-Å resolution. The two half-maps from the local reconstruction were used for sharpening in DeepEMhancer (47). The reported resolutions are based on the gold-standard criterion of a Fourier shell correlation of 0.143, and Fourier shell correlation curves were corrected for the effects of soft masking by high-resolution noise substitution (48, 49).

### Model building and refinement.

For the NTD, a published crystal structure (Protein Data Bank [PDB] accession number 7L2C) was docked into the locally refined and sharpened map in UCSF Chimera (50) and then manually fit using COOT (51). Previously unbuilt regions were manually built. N-linked glycans were built manually in COOT using the glyco extension, and their stereochemistry and fit to the map were validated with Privateer (52). For the Fab, the variable regions of the structures with the highest sequence identity to PVI.V6-14 (PDB accession numbers 6XWD and 5XI5 for the heavy and light chains, respectively) were docked into the focused map in UCSF Chimera and then used as a reference for manual building. For the remainder of the spike, a previously published structure (PDB accession number 7NTC) was docked into the sharpened full map in UCSF Chimera and then manually fit in COOT. All models were then refined in Phenix (53) using real-space refinement against their relative maps.

### Data availability.

All data are provided in the supplemental material. Requests for material should be addressed to Goran Bajic. The EM maps have been deposited in the Electron Microscopy Data Bank (EMDB) under accession numbers EMD-24402 and EMD-24403, and the accompanying atomic coordinates have been deposited in the Protein Data Bank (PDB) under accession numbers 7RBU and 7RBV.
