# Supplementary figures and images for: Structure of a Vaccine-Induced, Germline-Encoded Human Antibody Defines a Neutralizing Epitope on the SARS-CoV-2 Spike N-Terminal Domain
Source: mBio. 2022 Apr 25;13(3):e03580-21. doi: 10.1128/mbio.03580-21 (PMC9239078; doi:10.1128/mbio.03580-21)

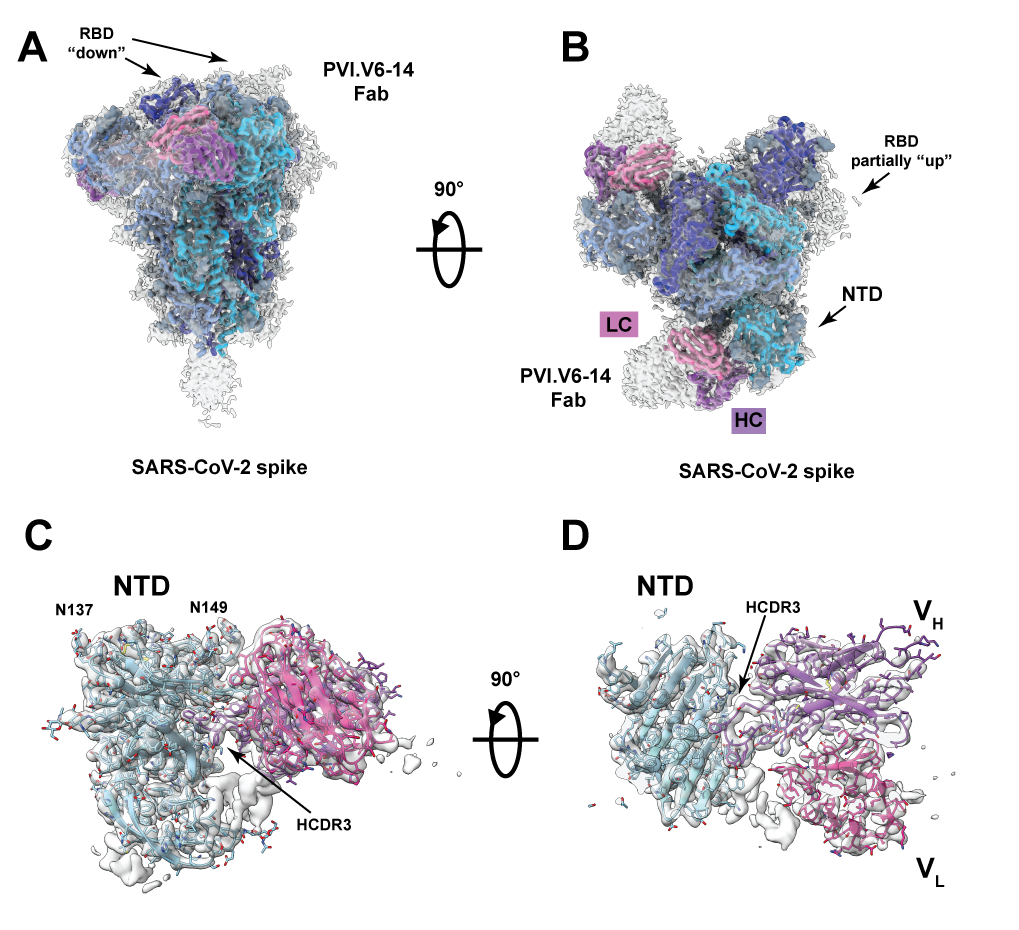

Supplement: FIG S1 [file mbio.03580-21-sf001.tif]

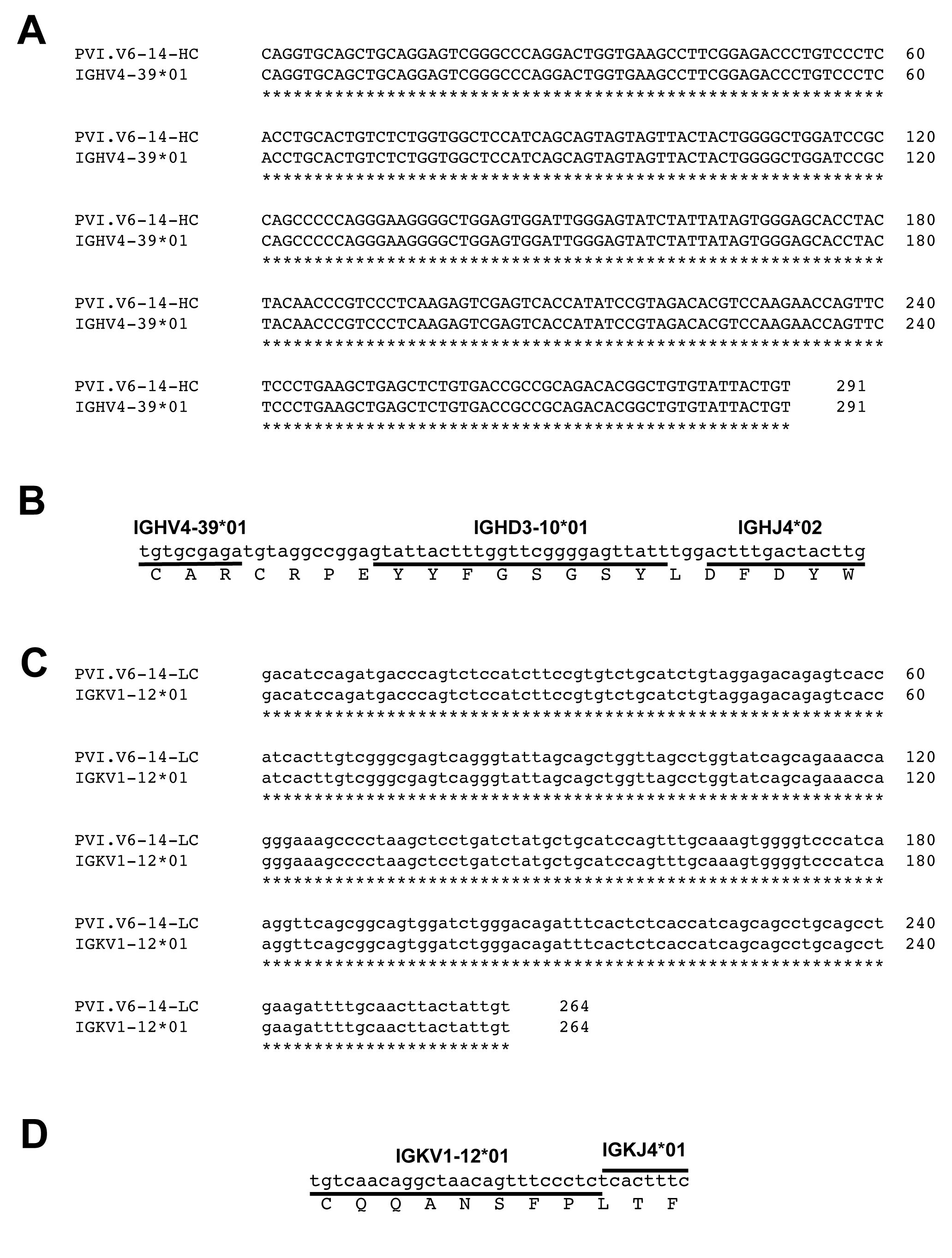

Supplement: FIG S2 [file mbio.03580-21-sf002.tif]

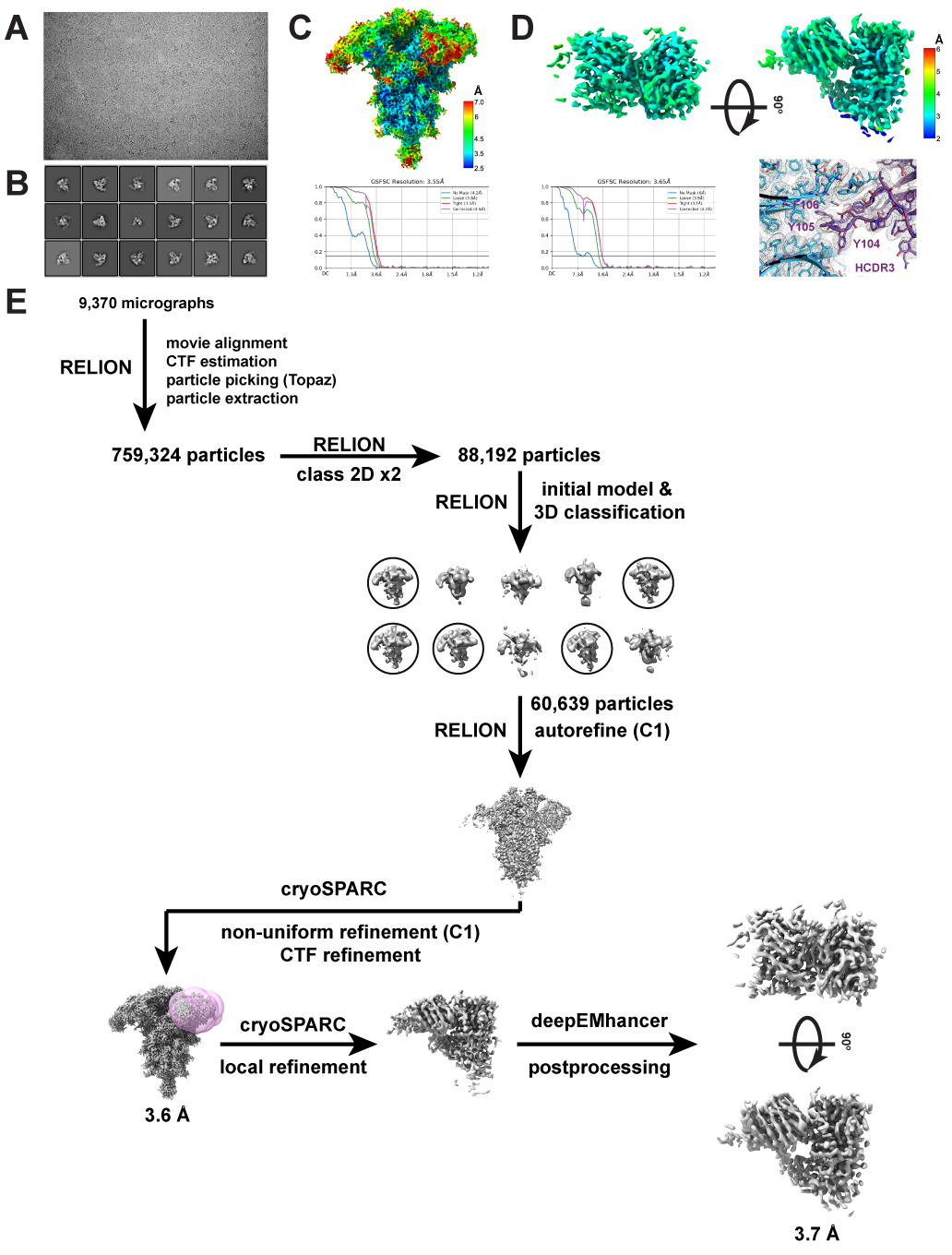

Supplement: FIG S3 [file mbio.03580-21-sf003.tif]

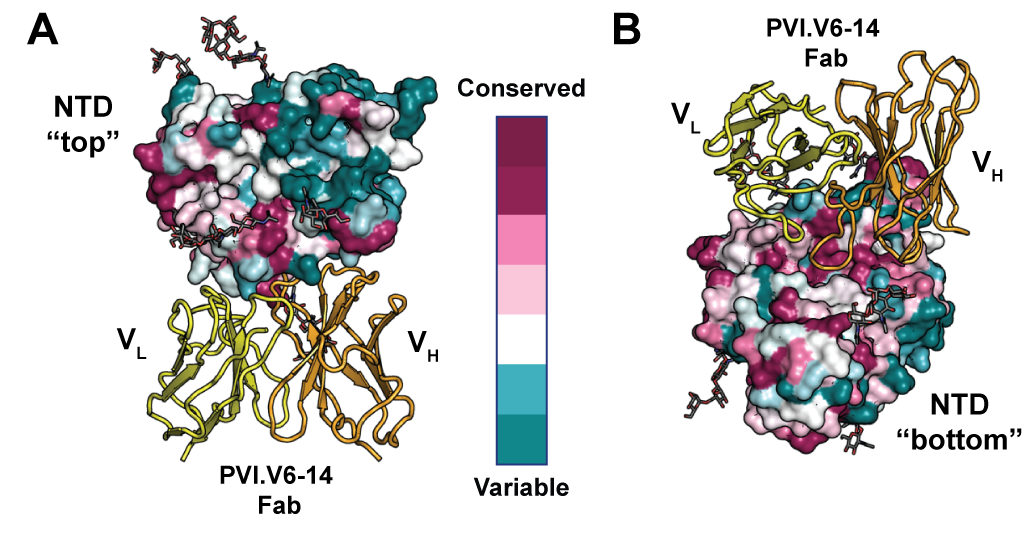

Supplement: FIG S4 [file mbio.03580-21-sf004.tif]
