# Supplementary material for: Structure of a Vaccine-Induced, Germline-Encoded Human Antibody Defines a Neutralizing Epitope on the SARS-CoV-2 Spike N-Terminal Domain
Source: mBio. 2022 Apr 25;13(3):e03580-21. doi: 10.1128/mbio.03580-21 (PMC9239078; doi:10.1128/mbio.03580-21)
Supplement: TABLE S1 [file mbio.03580-21-st001.docx]

| **Data collection** | | |
| --- | --- | --- |
| Grid type | **UltrAuFoil gold R0.6/1** |  |
| Microscope/voltage/detector | **Totan Krios/300 kV/Gatan K3 summit** |  |
| Magnification | **81,000** |  |
| Recording mode | **super-resolution counting mode** |  |
| Total dose | **50 e-/Å^2^/s** |  |
| Pixel size | **0.56** Å/pixel |  |
| Defocus range | **–2.5 to –0.8 μm** |  |
| No. micrographs used | **9,370** |  |
| Total particles picked | **759,324** |  |
| **Model validation** | | |
|  | **Spike:PVI.V6-14** | **NTD:PVI.V6-14** |
| Composition (#) |  |  |
| Chains | **7** | **4** |
| Atoms | **30431 (Hydrogens: 0)** | **4191** |
| Residues | **Protein: 3718** | **Protein: 511** |
| Water | **0** | **0** |
| Ligands | **BMA: 15**  **NAG: 78**  **MAN: 18** | **BMA: 1**  **NAG: 10**  **MAN: 2** |
| **Bonds (RMSD)** | | |
| Length (Å) (# > 4sigma) | **0.004 (0)** | **0.004 (0)** |
| Angles (°) (# > 4sigma) | **0.959 (3)** | **0.773 (4)** |
| MolProbity score | **1.87** | **1.97** |
| Clash score | **6.85** | **6.69** |
| **Ramachandran plot (%)** | | |
| Outliers | **0.19** | **0.2** |
| Allowed | **8.03** | **11.29** |
| Favored | **91.78** | **88.12** |
| Rotamer outliers (%) | **0.19** | **0.0** |
| Cbeta outliers (%) | **0.0** | **0.0** |
| Peptide plane (%) |  |  |
| Cis proline/general | **0.0/0.0** | **0.0/0.0** |
| Twisted proline/general | **0.0/0.0** | **0.0/0.0** |
| ADP (B-factors) min/max/mean | |  |
| Protein | **24.61/452.36/97.45** | **28.21/127.83/58.26** |
| Ligand | **54.74/450.94/189.42** | **45.97/161.64/96.72** |
| Water | **---** | **---** |
| **Data** | | |
| Unit cell info | | |
| Lengths (Å) | **151.76, 161.84, 166.88** | **91.28, 68.32, 81.76** |
| Angles (°) | **90.00, 90.00, 90.00** | **90.00, 90.00, 90.00** |
| Supplied Resolution (Å) | **3.6** | **3.7** |
| Resolution Estimates (Å) **Masked Unmasked** | | |
| d FSC model (0/0.143/0.5) | **2.1/2.3/3.7 2.1/2.3/3.8** | **3.5/3.6/3.9**  **3.6/3.7/4.0** |
| Map min/max/mean | **-0.04/3.04/0.04** | **-1.36/2.11/0.05** |
| **Model vs. Data** | | |
| CC (mask) | **0.74** | **0.81** |
| CC (box) | **0.72** | **0.66** |
| CC (peaks) | **0.61** | **0.57** |
| CC (volume) | **0.72** | **0.78** |
| Mean CC for ligands | **0.48** | **0.54** |
